# Association between night work and cardiovascular diseases: analysis of the 3rd Korean working conditions survey

**DOI:** 10.1186/s40557-015-0064-1

**Published:** 2015-05-11

**Authors:** Sungjin Park, Juhyun Nam, Jong-Ku Lee, Sung-Soo Oh, Hee-Tae Kang, Sang-Baek Koh

**Affiliations:** Department of Occupational and Environmental Medicine, Wonju Severance Christian Hospital, Wonju College of Medicine, Yonsei University, Wonju, South Korea

**Keywords:** Night work, Cardiovascular diseases, Korean working conditions survey

## Abstract

**Objectives:**

This study was conducted to investigate the relationship between night work and cardiovascular diseases among wage workers in Korea.

**Methods:**

The study was based on the 3rd Korean Working Conditions Survey (KWCS; 2011). This study included 29,711 wage workers. We used the chi-squared test and logistic regression to examine the association between cardiovascular diseases and night work and cumulative night work.

**Results:**

Among all of the paid workers, 12.5% reported doing night work ≥ 1 day per month. Night work was significantly associated with an increased risk of cardiovascular diseases (odds ratio [OR] 1.58, 95% confidence interval [CI] 1.11-2.25). Also, compared to the group that did not do night works, the group with higher cumulative night work demonstrated an increased risk for cardiovascular diseases (OR 1.81, 95% CI 1.19-2.74).

**Conclusions:**

This study suggests that night work is significantly associated with cardiovascular diseases.

## Introduction

Night work is currently common throughout the world. Shift work including a night work is being widely adopted in many places including China, the Republic of Korea, and other countries in Asia and Europe. In China, 36.1% of employees are shift workers, and in Korea more than 30% of those in the service industry do shift-based work [1]. In the EU-25, 17.7% of all employees do shift work [1]. Night work in the shift-based work system is thus also increasing accordingly. In Chile, Czechoslovakia, and China, 15-24% of employees work at night [2-4]. Some workers in Brazil (9.0%) and Hungary (9.5%) also do night work on a regular basis [5,6]. In the meantime, shift work including night work is known to be related with chronic diseases such as cardiovascular diseases, metabolic syndrome, diabetes, and breast cancer [7].

Shift work, in particular, is a risk factor for cardiovascular diseases. Wang et al. [7] noted, however, that the association between shift work including night work and cardiovascular diseases is controversial. Shift work has been reported to be linked with an increased risk for cardiovascular diseases in cohort studies [8,9]. The underlying mechanism includes changes in lifestyle factors caused by disruption of circadian rhythms and an increase in psychosocial stress. Shift workers have a reversed sleep-wake cycle, experiencing disrupted circadian rhythms. This leads to a higher stress level and adverse changes in lifestyle factors such as smoking and eating habits [10,11]. In addition, shift work is associated with an increase in cardiovascular diseases risk factors such as hypertension and obesity. The hike in the risk of those conditions due to shift work may also result in a higher risk for cardiovascular diseases [12,13].

In the meantime, few studies have examined the association between night work and cardiovascular diseases, or the risk factors for such diseases in Korean workers. Some studies have reported links between the period of shift work and risk factors for cardiovascular diseases —blood pressure, serum cholesterol level, Body Mass Index (BMI), Waist-Hip Ratio (WHR), and fasting glucose [13]. Furthermore, studies have been carried out on the association between shift work and cardiovascular symptoms [14] and between shift work and metabolic syndrome [15]. However, the subjects of those studies were limited to women [14,15], or had specific occupations such as nurses [13]. In other words, there has been no study using large-scale national data. Therefore, in this study, we intended to identify any association between night work and cardiovascular diseases based on large-scale national data representative of the population of the Republic of Korea as a whole.

## Materials and methods

### Study subjects

This study is based on the 3rd Korean Working Conditions Survey (KWCS) of 2011. The KWCS has been conducted since 2006, showing the current trends in working conditions, health, and occupational hazards of Korean employees. The 3rd KWCS was conducted from June 1, 2011 to November 30, 2011. The survey was administered to workers over 15 years old who had done paid work for more than one hour during the past week at the time of the survey. A total of 50,032 workers were surveyed. We restricted the population of study to paid workers, and thus 29,711 participants were included.

### General characteristics

The general characteristics collected in the KWCS included the following: sex; age (<30 years, 30-39 years, 40-49 years, 50-59 years, or ≥ 60 years); level of education completed (middle school or below, high school, or college or above); monthly income (<1 million won, 1 million–2 million won, 2 million–3 million won, or ≥ 3 million won); smoking status (non-smoker, ex-smoker, or current smoker); frequency of drinking alcohol (none, ≤ 1 drink per week, or ≥ 2 drinks per week); diagnosis of hypertension (yes or no); and diagnosis of obesity (yes or no). Hypertension and obesity were assessed with the question, “Have you ever been told by your doctor that you have (A) hypertension or (B) obesity?”

### Occupational characteristics

The occupational characteristics surveyed included the following: occupation type (white collar, service, or blue collar); company size by current number of employees (<5, 5-49, 50-299, or ≥ 300); years of service (<1 year, 1-9 years, ≥ 10 years); shift work status (yes or no); and working time (<40 hours a week, 40-48 hours a week, 49-60 hours a week, or ≥ 61 hours a week).

### Night work and cumulative night work

For this analysis, we defined night work as at least two hours of work between 10 pm and 5 am. Night workers were defined as those doing at least one night work within a month. Cumulative night work was calculated by multiplying the number of months worked by the number of night work done. Based on the mean (360), we categorized the subjects into a low risk group (those with < mean) and high risk group (those with ≥ mean). The analysis was performed using a group that had done no night work as the reference group.

### Cardiovascular diseases

To examine the subjects' history of cardiovascular diseases, the following question was asked: “Have you had any cardiovascular disease in the past 12 months?” Respondents answering "Yes" were considered to have cardiovascular diseases.

### Statistical analysis

We conducted a descriptive analysis on the general and occupational characteristics of the subjects surveyed. The crude odds ratio (OR) between cardiovascular diseases and night work and the 95% confidence interval (CI) were calculated using simple logistic regression analysis. In addition, the adjusted OR and 95% CI were calculated through multivariate logistic regression analysis after correcting for general and occupational characteristics. The analysis models according to the adjusted variables were as follows: (i) Model 1: crude OR; (ii) Model 2: adjusted for sex, age, level of education completed, and monthly income; (iii) Model 3: Model 2 + adjusted for smoking status, and frequency of drinking; (iv) Model 4: Model 3 + adjusted for occupation type, company size by the number of employees, shift work status, and working time. P ≤ 0.05 was considered to indicate statistical significance. The data was analyzed using SAS version 9.3.

## Results

### General characteristics of the study subjects

Among all paid workers, women and men accounted for 41.6% (12,365) and 58.4% (17,346) of the total, respectively. Those in their 30s were the most common (30.0%, 8,919), followed by those in their 40s (28.7%, 8,534), 50s (16.9%, 5,019), under 30 (16.5%, 4,904), and 60 and older (7.9%, 2,335). Concerning the level of education completed, those with college or above comprised the largest proportion (49.8%, 14,791), followed by those with a high school diploma (39.0%, 11,600), and middle school or below (11.2%, 3,320). Among monthly income levels, the largest group had an income in the range of 1-2 million won (39.8%, 11,825), followed by 2-3 million won (27.1%, 8,058), ≥ 3 million (20.3%, 6,041), and < 1 million won (12.7%, 3,780). Concerning the participants’ smoking status, 54.9% (16,318) were non-smokers, while 34.1% (10,127) were current smokers and 11.0% (3,266) were ex-smokers. Among those surveyed, 51.0% (15,153) drank ≤ 2 alcoholic drinks a week, while 25.7% (7,634) drank < 1 drink a week. Non-drinkers represented 23.3% (6,924) of the total. There were 1,463 (4.9%) who had been diagnosed with hypertension and 553 (1.9%) diagnosed with obesity. When asked if they had experienced cardiovascular diseases in the past year, 1.1% (335) responded affirmatively.

With regard to occupational characteristics, white collar workers were the largest group, constituting 12,149 (40.9%), while 10,735 were blue collar workers and 6,827 were service workers. As for the size of the workplace, 50.2% (14,478) were working at a site with 5-49 people; 26.3% (7,591) belonged to a site with < 5 workers; 16.4% (4,724) worked at a site with 50-299 workers; 7.1% (2,052) had a workplace with ≥ 300 employees. Concerning the years of service, 60.9% (18,091) reported working for 1-9 years; 23.9% (7,010) had ≥ 10 years’ experience; and 15.2% (4,519) had worked for < 1 year. Shift workers accounted for 9.6% (2,839), and the rest (26,872; 90.4%) were not shift workers. With regard to work hours, 52.2% (15,521) worked for 40–48 hours a week, 28.9% (8,571) worked for 49–60 hours a week, 10.0% (2,970) worked < 40 hours a week, and 8.9% (2,649) worked for ≥ 61 hours a week. Those who said they did night work at least once a month constituted 12.5% (3,712), and the rest 87.5% (25,999) said that they did not (Table 1).

**Table 1:** **General characteristics of the study subjects**

	**N**	**%**
Sex		
Male	17346	58.4
Female	12365	41.6
Age (years)		
<30	4904	16.5
30-39	8919	30.0
40-49	8534	28.7
50-59	5019	16.9
≥60	2335	7.9
Level of education completed		
Middle school or below	3320	11.2
High school	11600	39.0
College or above	14791	49.8
Monthly income (Korean won)		
<1,000,000	3780	12.7
1,000,000 – 2,000,000	11825	39.8
2,000,000 – 3,000,000	8058	27.1
≥3,000,000	6041	20.3
Smoking		
Non-smoker	16318	54.9
Ex-smoker	3266	11.0
Current smoker	10127	34.1
Alcohol drinking frequency (drinks)		
None	6924	23.3
≤1 per week	7634	25.7
≥2 per week	15153	51.0
Job type		
White collar	12149	40.9
Service	6827	23.0
Blue collar	10735	36.1
Company size (no. of employees)		
<5	7591	26.3
5-49	14478	50.2
50-299	4724	16.4
≥300	2052	7.1
Years of service		
<1	4519	15.2
1-9	18091	60.9
≥10	7101	23.9
Shift work		
No	26872	90.4
Yes	2839	9.6
Working time (hours/week)		
<40	2970	10.0
40-48	15521	52.2
49-60	8571	28.9
≥61	2649	8.9
Hypertension		
No	28248	95.1
Yes	1463	4.9
Obesity		
No	29158	98.1
Yes	553	1.9
Cardiovascular diseases		
No	29376	98.9
Yes	335	1.1
Night work		
No	25999	87.5
Yes (≥1 day/month)	3712	12.5

### Association between general characteristics and night work

Among the subjects who were night workers, the males comprised a higher proportion (77.4%) of the total than females, and those in their 50s and 60s took up larger shares of the total, while those under 30, and those in their 30s and 40s each constituted lower proportions. Those with high school education and education below middle school accounted for more of the night workers, whereas those with a college education or above appeared less frequently among night workers. Regarding average monthly income, subjects with an income between 2-3 million won and ≥ 3 million were more common among night workers; those with < 1 million won accounted for a smaller proportion. Concerning the smoking status, there were more current smokers and ex-smokers among night workers (44.3% and 16.4%, respectively) than among workers without night work (32.6% and 10.2%). The proportions of those who drink ≥ 2 alcoholic drinks per week and those who do not drink at all were smaller among night workers than in the other workers, while the share of those who drink ≤ 1 per week was higher in the night workers. There were more who had been diagnosed with hypertension in the night worker group than among the other workers (6.9% and 4.6%, respectively). There were also more who had been diagnosed with obesity among the night workers than in rest of the workers (2.8% and 1.7%, respectively). The share of those who said they had experienced cardiovascular diseases was larger among the night workers (1.8%) than in those who did not work night work (1.0%). All of the aforementioned variables showed statistically significant differences.

Among the subjects, there were more blue collar workers among the night workers (52.0%) than in the other workers (33.9%), whereas white collar workers and service workers were fewer in the night workers. The subjects who work for sites with 50-299 employees and sites with ≥ 300 workers were more common in night workers (22.8% and 14.1%, respectively) than workers without night work (15.5% and 6.1%, respectively). The shares of workers who belong to sites with < 5 employees and 5-49 employees were smaller in the night worker group. Concerning the years of service, there were more who had worked ≥ 10 years in the night workers, and there were fewer of those who had worked < 1 year or had worked for 1-9 years among the night workers. A much greater proportion of night workers (50.6%) than the other workers (3.7%) were involved in shift work. The shares of those who work for 49-60 hours and for ≥ 61 hours were higher in the night workers (32.8% and 24.8%, respectively) than non-night workers (28.3% and 6.6%, respectively), while the proportion of those who work < 40 hours or 40-48 hours a week were lower in the night workers. All of the aforementioned variables showed statistically significant differences (Table 2).

**Table 2:** **Association between general characteristics and night work**

	**Night work**				p value*
	**Yes** **(N** ** = 3712)**		**No** **(N = ** **25999)**		
	**N**	**%**	**N**	**%**	
Sex					
Male	2872	77.4	14474	55.7	<.0001
Female	840	22.6	11525	44.3	
Age (years)					
<30	596	16.1	4308	16.6	<.0001
30-39	1025	27.6	7894	30.4	
40-49	941	25.4	7593	29.2	
50-59	669	18.0	4350	16.7	
≥60	481	13.0	1854	7.1	
Level of education completed					
Middle school or below	492	13.3	2828	10.9	<.0001
High school	1683	45.3	9917	38.1	
College or above	1537	41.4	13254	51.0	
Monthly income (Korean won)					
<1,000,000	364	9.8	3416	13.1	<.0001
1,000,000 – 2,000,000	1421	38.3	10404	40.0	
2,000,000 – 3,000,000	1070	28.8	6988	26.9	
≥3,000,000	857	23.1	5184	19.9	
Smoking					
Non-smoker	1460	39.3	14858	57.2	<.0001
Ex-smoker	608	16.4	2658	10.2	
Current smoker	1644	44.3	8483	32.6	
Alcohol drinking frequency (drinks)					
None	677	18.2	6247	24.0	<.0001
≤1 per week	1179	31.8	6455	24.8	
≥2 per week	1856	50.0	13297	51.1	
Job type					
White collar	1023	27.6	11126	42.8	<.0001
Service	758	20.4	6069	23.3	
Blue collar	1931	52.0	8804	33.9	
Company size (no. of employees)					
<5	627	17.4	6964	27.6	<.0001
5-49	1634	45.3	12844	50.9	
50-299	822	22.8	3902	15.5	
≥300	523	14.5	1529	6.1	
Years of service					
<1	539	14.5	3980	15.3	0.0004
1-9	2190	59.0	15901	61.2	
≥10	983	26.5	6118	23.5	
Shift work					
No	1833	49.4	25039	96.3	<.0001
Yes	1879	50.6	960	3.7	
Working time (hours/week)					
<40	197	5.3	2773	10.7	<.0001
40-48	1377	37.1	14144	54.4	
49-60	1216	32.8	7355	28.3	
≥61	922	24.8	1727	6.6	
Hypertension					
No	3457	93.1	24791	95.4	<.0001
Yes	255	6.9	1208	4.6	
Obesity					
No	3607	97.2	25551	98.3	<.0001
Yes	105	2.8	448	1.7	
Cardiovascular diseases					
No	3647	98.2	25729	99.0	<.0001
Yes	65	1.8	270	1.0	

*based on a chi-squared test.

### Odds ratios of cardiovascular diseases associated with night work

Night work was found to have a significant association with cardiovascular diseases. When the group that does night work ≥ 1 day per month and the group which does not were compared, the crude OR and 95% CI of night work was 1.70 (1.29-2.23). The OR and 95% CI after correcting general and occupational characteristics were as follows: Model 2 (OR 1.44, 95% CI 1.09-1.92); Model 3 (OR 1.42, 95% CI 1.07-1.89); Model 4 (OR 1.58, 95% CI 1.11-2.25) (Table 3).

**Table 3:** **Odds ratios of cardiovascular disease associated with night work**

	**Model 1**		**Model 2**		**Model 3**		**Model 4**	
	**OR**	**95%** **CI**	**OR**	**95%** **CI**	**OR**	**95%** **CI**	**OR**	**95%** **CI**
Cardiovascular diseases								
Reference group	1.00		1.00		1.00		1.00	
Night work (≥1 day/month)	1.70	1.29 - 2.23	1.44	1.09 - 1.92	1.42	1.07 - 1.89	1.58	1.11 - 2.25

OR, odds ratio; CI, confidence interval.

Model 1: odds ratio by independent univariate logistic regression analysis.

Model 2: multivariate logistic regression analysis, model 1 + gender (male or female), age (<30 years, 30-39 years, 40-49 years, 50-59 years, or ≥60 years, level of education completed (middle school or below, high school, or college or above), monthly income (<1,000,000 won, 1,000,000 – 2,000,000 won, 2,000,000 – 3,000,000 won, or ≥3,000,000 won).

Model 3: multivariate logistic regression analysis, model 2 + smoking (non-smoker, ex-smoker or current smoker), and drinking frequency (none, ≤1 per week, ≥2 per week).

Model 4: multivariate logistic regression analysis, model 3 + job type (white collar, service, or blue collar), company size by number of employees (<5, 5-49, 50-299, or ≥300), shift work (yes or no), and working time (<40 hours a week, 40-48 hours a week, 49-60 hours a week, or ≥61 hours a week).

### Odds ratios of cardiovascular diseases associated with cumulative night work

We measured cumulative night work by multiplying the number of night work done per month by the number of months worked. We divided the participants into low-risk and high-risk groups based on the mean, and compared them with the reference group which did not do night work, and analyzed the results. There was no significant increase in the risk of cardiovascular diseases in the low risk group compared to the reference group (Model 1: Crude OR 1.26, 95% CI 0.82-1.93; Model 2: OR 1.25, 95% CI 0.81-1.94; Model 3: OR 1.23, 95% CI 0.79-1.90; Model 4: OR 1.34, 95% CI 0.84-2.14). The high risk group demonstrated a significant increase in the risk in all models compared to the reference group (Model 1: Crude OR 2.10, 95% CI 1.51-2.92; Model 2: OR 1.58, 95% CI 1.12-2.22; Model 3: OR 1.56, 95% CI 1.11-2.20; Model 4: OR 1.81, 95% CI 1.19-2.74) (Table 4).

**Table 4:** **Odds ratios of cardiovascular disease associated with cumulative night work**

	**Model 1**		**Model 2**		**Model 3**		**Model 4**	
	**OR**	**95%** **CI**	**OR**	**95%** **CI**	**OR**	**95%** **CI**	**OR**	**95%** **CI**
Cardiovascular diseases								
Reference group	1.00		1.00		1.00		1.00	
Low risk group (<360)	1.26	0.82 - 1.93	1.25	0.81 - 1.94	1.23	0.79 - 1.90	1.34	0.84 - 2.14
High risk group (≥360)	2.10	1.51 - 2.92	1.58	1.12 - 2.22	1.56	1.11 - 2.20	1.81	1.19 - 2.74

OR, odds ratio; CI, confidence interval.

Model 1: odds ratio by independent univariate logistic regression analysis.

Model 2: multivariate logistic regression analysis, model 1 + sex (male or female), age (<30 years, 30-39 years, 40-49 years, 50-59 years, or ≥60 years, level of education completed (middle school or below, high school, or college or above), monthly income (<1,000,000 won, 1,000,000 – 2,000,000 won, 2,000,000 – 3,000,000 won, or ≥3,000,000 won).

Model 3: multivariate logistic regression analysis, model 2 + smoking (non-smoker, ex-smoker or current smoker), and drinking frequency (none, ≤1 per week, ≥2 per week).

Model 4: multivariate logistic regression analysis, model 3 + job type (white collar, service, or blue collar), company size by number of employees (<5, 5-49, 50-299, or ≥300), shift work (yes or no), and working time (<40 hours a week, 40-48 hours a week, 49-60 hours a week, or ≥61 hours a week).

## Discussion

This study was conducted to search for an association between night work and cardiovascular diseases in Korean paid workers, using large-scale national data representative of the population. Among all paid employees, those who did a night work ≥ 1 day per month accounted for 12.5%. In the group that did night work, the OR (95% CI) of cardiovascular diseases was 1.58 (1.11-2.25), suggesting a significant association with an increase in the risk of cardiovascular diseases. We speculated that the number of night work done would raise the risk for cardiovascular diseases. For verification, we calculated the cumulative night work, which showed a higher risk of cardiovascular diseases in the group who had greater cumulative night work.

A prior study—a cross-sectional one in Brazil using the Framingham score as an indicator of cardiovascular risk—showed that night shift work was independently associated with an increase in cardiovascular risk [16]. In addition, the hazard ratio of myocardial infarction in German adults doing shift work was 1.53 (95% CI 1.06-2.22) [17]. Literature reviews have revealed a significant association between shift work including night work and cardiovascular diseases [7,11,18]. Cohort studies have also reported significant links between shift work and cardiovascular diseases. According to an observational study following 5,517 Danish workers for 12 years, the relative risk of circulatory disease in nighttime workers compared to daytime workers was 1.31 (95% CI 1.06-1.63) [8]. Another cohort study in which 6,711 Japanese workers were followed for 14 years demonstrated that shift workers had a higher risk of mortality due to ischemic heart disease than daytime workers (relative risk = 2.32, 95% CI 1.37-3.95) [9]. Such results agree with those of this study. Nonetheless, as the definition of cardiovascular diseases and night work and the measurement methods of night work differ in various studies, caution is needed in interpreting the results.

Studies that have analyzed the dose-response relationship between night shift work and cardiovascular diseases include those of Brown et al. (2009) [19] and Kawachi et al. (1995) [20]. Brown et al. reported in their cohort study using the Nurses’ Health Study, that with every five years of rotating night shift work, the risk of ischemic stroke went up by 4% (HR = 1.04, 95% CI 1.01-1.07)[19]. Kawachi et al., also using the Nurses’ Health Study dataset, stated that the relative risk of coronary heart disease of a group with 6 or more years of rotating night shifts was 1.51 (95% CI 1.12-2.03), compared to the group which did not do shift work [20]. The number of months worked, as used in this study, is not the exact period of the subjects’ night work. Therefore, it is not reasonable to compare the risk of cardiovascular diseases with the number of night work done per month alone. Also, as younger and healthier people are more likely to do shift work [11,21], the period of shift work had to be taken into consideration. Therefore, we calculated and analyzed cumulative night work. Consequently, the risk of the low-risk group compared with the group that did not have cumulative night work was not significantly different (OR = 1.34, 95% CI 0.84-2.14). In contrast, the risk of cardiovascular diseases in the high risk group was higher than in the reference group (OR 1.81, 95% CI 1.19-2.74).

Various biological mechanisms behind the link between shift work and cardiovascular diseases have been suggested. Disruption of circadian rhythms, changes in metabolic and hormonal functioning, lifestyle changes including smoking and unhealthy eating habits, and psychosocial stress are some of the examples [7,10,11,18]. Night workers have a polar opposite schedule for work and rest from the standard chronobiological pattern. They sleep when the body is preparing to start activity and have to work when their psychophysical activity is decreased [22]. Such changes in the chronobiological pattern in the human body disrupt the normal circadian rhythms of blood pressure, which drops at night and increases in the morning [22]. In other words, shift work converts the changes of blood pressure in a day from a dipper to non-dipper pattern [23,24]. "Dipper" here refers to the drop of nighttime blood pressure by more than 10% of the average mean arterial pressure. “Non-dipper” means no or minimal drop of the nighttime blood pressure [24]. Such a limited decrease in the nighttime blood pressure due to circadian rhythm disruption (non-dipping pattern) has been reported to increase the risk of cardiovascular diseases [22,25].

In this study, while we did not measure the blood pressure of the subjects, we instead surveyed whether they had been clinically diagnosed with hypertension. In the results, the proportion of those who had been diagnosed with hypertension was higher in the night workers (6.9%) than non-night workers (4.6%). Another study revealed an association between night shift work and hypertension. In a study on 1,838 female workers in one Taiwanese manufacturing company, women who did a night shift showed a significantly higher OR of hypertension than their daytime counterparts (OR 2.30, 95% CI 1.20-4.40) [26].

In the meantime, Tenkanen et al. reported that risk factors for coronary artery disease such as smoking and obesity facilitated physiologic and metabolic disturbances by circadian rhythm disruption following shift work [27]. According to the study, shift work reduces fibrinolytic activity and increases plaque formation. As smoking and obesity reduce fibrinolysis and increase the fibrinogen level and ability of platelets to aggregate, they accelerate the adverse process caused by night work. Meanwhile, the smoking rate is higher in shift workers than day time workers [9]. According to the results of this study, the proportions of current smokers and former smokers were higher in the night workers (44.3% and 16.4%, respectively) than in the other workers (32.6% and 10.2%, respectively). Shift work is associated with an increase in BMI, body weight, and comorbidity of obesity [28]. Although we did not measure BMI in this study, the percentage of those who were diagnosed with obesity was higher in the night workers (2.8%) than in workers who did not do night work (1.7%).

Studies on shift work and cardiovascular diseases have also been conducted in Korea. Ha and Park demonstrated an association between the duration of shift work and metabolic risk factors of cardiovascular diseases in a study on 226 female nurses and 134 male employees in a manufacturing company. The researchers found that an increase in shift work duration leads to a statistically significant increase in blood pressure, serum cholesterol level, and WHR [13]. In addition, Lee and Kim found significant increases in the cardiovascular and digestive symptoms in 1,875 female shift workers [14]. In a more recent study, Ye et al. analyzed 254 female workers and suggested that the OR of metabolic syndrome in shift workers was as high as 6.30 (95% CI 1.24-32.15) [15]. Nevertheless, the above studies had a small subject size, or focused only on a specific sex or occupation types.

This study has several limitations. First, being a cross-sectional study, it is difficult to determine causal relationship between variables through this study. Second, as the study was based on a survey, we were not able to collect objective measures such as blood pressure, BMI, and serum cholesterol. Moreover, information about cardiovascular diseases, night work and other general and occupational characteristics was obtained by self-report survey, which might lead to a possibility for recall bias. Finally, to calculate cumulative night work, we multiplied the number of night work done by the number of months worked. However, as the number of months worked is not exactly same as the duration of night work, cumulative night work may not have produced accurate estimates.

In spite of these limitations, this study is important in that this study is an epidemiologic study that identified an association between cardiovascular diseases and night work with adjustment for several possible confounders using national survey data representative of the Korean population.

## Conclusions

Overall, our data showed an increased risk of cardiovascular diseases among paid workers who did night work, and higher cumulative night work demonstrated an increased risk of cardiovascular diseases. But the results might have recall bias and should be interpreted with caution because the exposure variable and independent variables were collected from self-reported survey.

For future research, prospective studies are needed to demonstrate the causal association between the variables. In addition, an analysis specific to night work with more detailed variables (e.g., duration, intensity, direction, and type of shift work) is necessary.
